# Inhibition of HIV-1 assembly by coiled-coil domain containing protein 8 in human cells

**DOI:** 10.1038/srep14724

**Published:** 2015-10-01

**Authors:** Min Wei, Xia Zhao, Mi Liu, Zhi Huang, Yong Xiao, Meijuan Niu, Yiming Shao, Lawrence Kleiman

**Affiliations:** 1 School of Medicine, Nankai University, Tianjin, China, 300071; 2 Lady Davis Institute, Jewish General Hospital, McGill University, Canada, H3T 1E2; 3 State Key Laboratory for Infectious Disease Prevention and Control, National Center for AIDS/STD Control and Prevention, Chinese Center for Disease Control and Prevention, Beijing, China; 4 Peking University Health Science Center, Beijing, China

## Abstract

Human Immunodeficiency Virus type 1 (HIV-1) major structure protein Gag is synthesized in the cytoplasm, assembles on the plasma membrane, subsequently buds and releases. HIV-1 viral particles incorporate a number of host proteins to facilitate or inhibit HIV-1 replication. Here we identify a new host protein, coiled-coil domain containing protein 8 (CCDC8), in HIV-1 particles. Incorporation of CCDC8 into virions is dependent on the interaction between CCDC8 and Gag matrix region. Exogenous overexpression of CCDC8 can strongly inhibit HIV-1 production, up to ~30 fold. CCDC8 is a membrane-associated protein. The interaction between exogenously expressed CCDC8 and Gag on the plasma membrane changes the assembly of Gag, and redirects it into intracellular sites, or causes Gag endocytosis. CCDC8, along with cytoskeleton protein obscuring-like1 (Obsl1) and E3 ligase Cul7, induces Gag polyubiquitination and degradation. Thus we identify a new host protein and a new pathway for HIV-1 Gag polyubiquitination and degradation. This pathway presents potential therapeutic strategies against HIV infection.

Human immunodeficiency virus type 1 (HIV-1), the pathogen of AIDS, is a complex retrovirus and encodes structure proteins Gag, GagPol, Env, and regulatory proteins Vif, Vpu, Vpr, Tat, Nef, and Rev^1^. Expression of viral proteins is dependent on the viral mRNA export from the nucleus to the cytoplasm in host cells, which are facilitated by Rev and cis-acting element RRE (Rev-responsive element)^1^,^2^. Gag protein is the major structural protein of HIV-1 particles and Gag alone can form noninfectious virus-like particles (VLPs) without incorporation of viral RNA^1^. Gag proteins are synthesized as precursor p55 Gag in the cytoplasm, directed to membrane for assembly and processed by the viral protease during or shortly after budding to generate the mature Gag proteins matrix (MA, p17), capsid (CA, p24), nucleic capsid (NC, p7) and p6^1^.

To establish a productive infection in host cells, HIV-1 employs a number of host proteins to facilitate its replication, such as Alix and Tsg101, both of which can be found in the viral particles^3^,^4^,^5^,^6^. On the other hand, host cells also incorporate restriction factors into viral particles to limit HIV-1 replication, for example cytidine deaminase APOBEC3G (apolipoprotein B mRNA-editing, enzyme-catalytic, polypeptide-like 3G)^7^. However, the functions of dozens of virion-associated host proteins are unknown. Here we identified a new membrane-associated host protein, coiled-coil domain containing protein 8 (CCDC8) in the HIV-1 particles. Incorporation of CCDC8 into virions is mediated through the interaction between CCDC8 and Gag matrix region. Moreover, exogenous overexpression of CCDC8 strongly reduces HIV-1 Gag production and infectious virion release.

Mutations in CCDC8 have been reported to be associated with 3-M syndrome, an autosomal recessive primordial growth disorder characterized by severe pre- and postnatal growth retardation^8^,^9^,^10^,^11^. Genetic studies revealed that in patients with 3-M syndrome, approximately 70% have E3 ligase Cul7 mutations, 25% have cytoskeleton protein obscuring-like1 (Obsl1) mutations, and 5% have CCDC8 mutations^11^. Thus, 3-M syndrome links CCDC8, Obsl1 and Cul7, suggesting they could be in the same pathway. In this study, immunoprecipitation assay proves the interaction between Gag and CCDC8, and between CCDC8 and Obsl1 and Cul7. Furthermore, exogenously expressed CCDC8 causes Gag polyubiquitination and degradation. Therefore, we identified a new pathway for Gag polyubiquitination and degradation, although the significance of this pathway is unknown.

## Results

### Exogenous expression of CCDC8 strongly inhibits HIV-1 production

**Figure 1:**
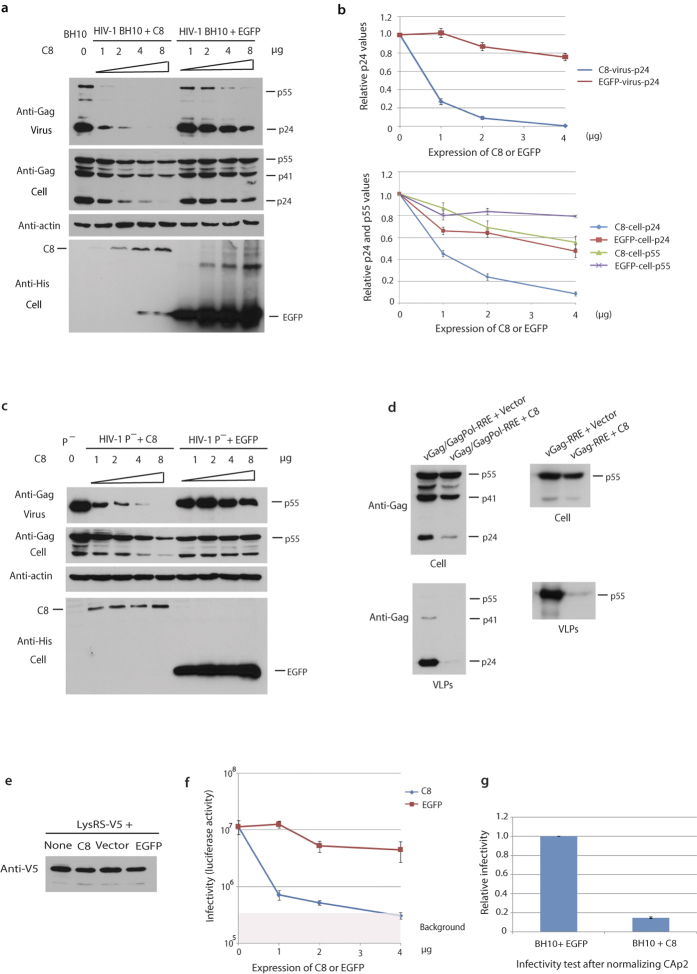
Inhibition of HIV-1 by CCDC8. (**a**) HEK293T cells were co-transfected with constant HIV-1 BH10 (2 μg) and increasing amounts of His-tagged pTT5-SH5-CCDC8 (C8) plasmid (at 0, 1, 2, 4, 8 μg) or negative control His-tagged pTT5-SH5-EGFP (Enhanced Green Fluorescent Protein, at 1, 2, 4, 8 μg). Lysates of virus and cells were monitored by Western blot, and probed with anti-Gag-CAp24, anti-β-actin, and anti-His antibodies. (**b**) CAp24 and Gag precursor p55 in the lysates of virus and cells were plotted based on the data of panel (**a**). The points of 8 μg plasmid were discarded. Error bar represents value ranges from the three experiments. (**c**) HIV-1 BH10 is replaced with protease negative HIV-1 BH10 P^−^ to repeat the experiment in panel (**a**). (**d**) Co-transfection of vGag/GagPol-RRE (left panel) or vGag-RRE (right panel) plus Rev with either empty vector or CCDC8 (C8) in HEK293T cells. CAp24 and Gag precursor p55 were monitored by Western blot. (**e**) V5 tagged lysyl-tRNA synthetase (LysRS) was co-transfected with nothing (none), CCDC8 (C8), empty vector (Vector) or EGFP (EGFP). Cell lysate was detected by Western blot with the probe anti-V5. (**f**) Infectious virus yield was measured using Hela-TZMbl indicator cells and expressed as luciferase activity (±standard error, n = 3). Pink box stands for the background of luciferase activity. (**g**) Relative infectivity was measured after normalizing CAp24 (2 ng).

Using mass spectrometry (MS), we analyzed proteins found in HIV-1 VLPs viral Gag (vGag-RRE), or protease-negative viral Gag/GagPol-RRE-P- (vGag/GagPol-P-), which produces unprocessed Gag and GagPol. Proteins present in each section of the gel were identified by MS. A dozen of cDNAs to identified proteins of unknown function were synthesized, and cloned into mammalian expression vector pTT5-SH5. HIV-1 Gag CAp24 production was measured upon the expression of above cDNA clones. During screening, only one of these proteins, a low-abundant species identified as coiled-coil domain containing protein 8 (CCDC8), can strongly inhibit HIV-1 CAp24 production. To further characterize the inhibition of HIV-1 production by CCDC8, we co-expressed constant amount of HIV-1 BH10 plasmid (2 μg) and increasing amounts of plasmid coding for His-tagged pTT5-SH5-CCDC8 in HEK293T cells, and followed by Western blot analysis. We found a drastic reduction in the amount of virus produced upon exposure to CCDC8, compared with a slower rate of reduction in the negative control (Enhanced Green Fluorescent Protein [pTT5-SH5-EGFP] expression) (Fig. 1a,b). This is accompanied by a mild reduction in cellular Gag production, but a much greater reduction in cellular CAp24, suggesting that Gag proteolytic processing could be impaired or Gag degradation could be increased or both. At lower concentrations of transfected plasmid of CCDC8, such as 1–4 μg, Gag expression is less affected and the extracellular CAp24 is reduced up to ~10–30 fold (Fig. 1a,b). In contrast, Gag and cell CAp24 are much less affected by expression of EGFP, or other cloned host proteins, such as milk-fat globule epidermal growth factor (EGF) factor 8 (MFG-E8), Zinc-alpha-2- glycoprotein (AZGP1), etc. (unshown data).

The experiments described were repeated using a protease-negative virus (HIV-1 BH10 P^−^). Again, co-expression of CCDC8 with HIV-1 BH10 P^−^ resulted in a very strong reduction in virus production accompanied by a milder reduction in cellular Gag (Fig. 1c). This data indicate that the inhibition of HIV-1 production by CCDC8 is independent on viral protease activity. Thus, the rapid reduction in CAp24 seen in Fig. 1a,b is probably not the primary factor responsible for the decrease in viral production, but could reflect a reduction in Gag proteolytic processing or an increase in Gag degradation. Meanwhile, we found that 8 μg plasmid of CCDC8 or an HIV-1/CCDC8 ratio at 1:4 in the transient transfection inhibited the expression of Gag (Fig. 1a,c). Thus, in the later experiments, we used 1–4 μg plasmid of CCDC8 or a Gag/CCDC8 ratio at 1:1 or 1:2.

Moreover, without affecting expression of Gag, CCDC8 also inhibits vGag/GagPol-RRE or vGag-RRE production, which encodes only viral Gag/GagPol or viral Gag without any regulatory proteins (Fig. 1d). This data support the notion that the inhibition of HIV-1 production by CCDC8 is independent on viral protease activity. Meanwhile, the CCDC8 inhibition efficiency for vGag/GagPol-RRE is comparable to the HIV-1 whole genome containing plasmid BH10 (Fig. 1a–d), suggesting that HIV-1 does not encode regulatory proteins to antagonize CCDC8. It is unlike APOBEC3G, which activity is antagonized by HIV-1 regulatory protein Vif 7.

Furthermore, CCDC8 has no effect upon the production of β-actin (Fig. 1c) or the production of exogenous V5-tagged lysyl-tRNA synthetase (Fig. 1e). These data suggest that the effect of exogenous overexpression of CCDC8 upon extra-cellular virus production is specific.

HIV-1 inhibition by CCDC8 was also repeated in other cell lines, such as Hela cells. However, the inhibitory effect of CCDC8 overexpression on HIV-1 production was less effective in Hela cells than in HEK293T cells (unshown data).

### CCDC8 also reduces viral infectivity

Viral infectivity upon the overexpression of CCDC8 was measured in Hela-TZMbl indicator cell line. Either the same volume of virus-containing culture supernatant or the same amount of CAp24 was used to infect Hela-TZMbl cells. When the same volume of viral supernatant was used, infectivity of HIV-1 upon the overexpression of CCDC8 was reduced >17 fold, compared to the negative control EGFP (Fig. 1f). This will partially reflect a reduction in production of extracellular viral particles or reduced viral infectivity per particle. Indeed, upon infection of cells with normalized amount of CAp24 (2 ng), we found that infectivity per particle was still reduced by 6–7 fold (Fig. 1g).

### CCDC8 interacts with HIV-1 Gag matrix

**Figure 2:**
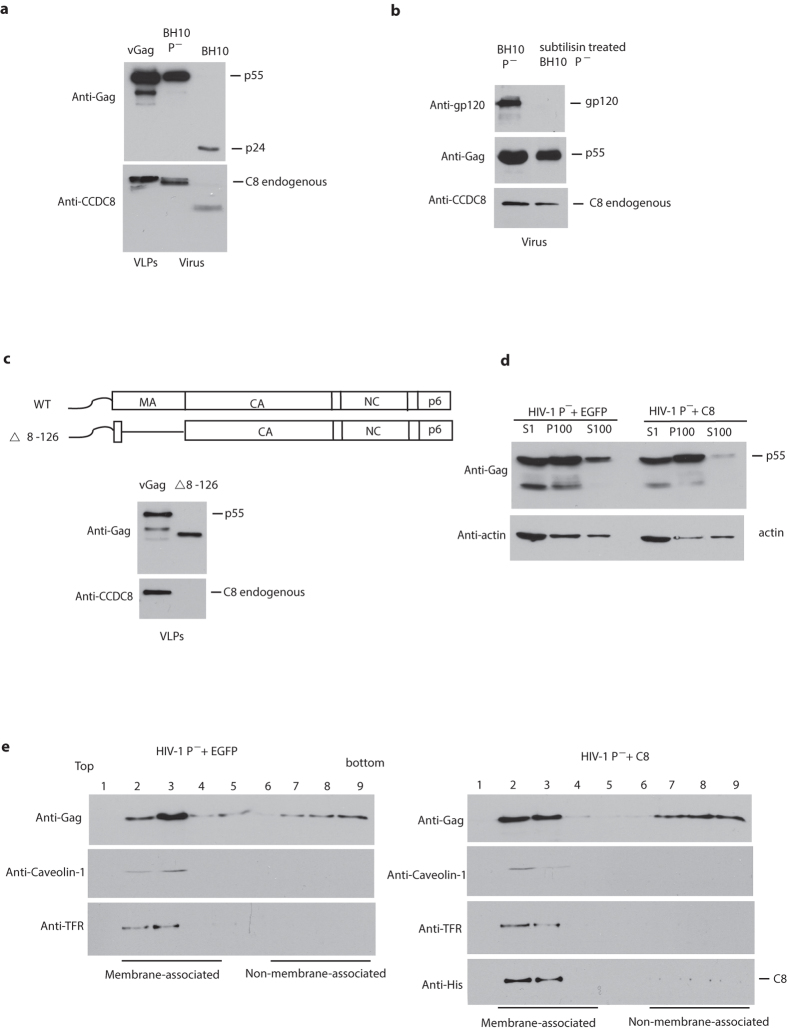
Incorporation of endogenous CCDC8 into HIV-1 virions or virus-like particles (VLPs) and CCDC8 is acting on the post-translational step of HIV-1 replication. (**a**) Virus CAp24 and endogenous CCDC8 were monitored by Western blot with anti-Gag-CAp24 or anti-CCDC8 antibodies after transfection with HIV-1 BH10, or P^−^, or vGag-RRE plus Rev in HEK293Tcells. (**b**) Pelletable HIV-1 BH10 P^−^ virions were treated or not treated with protease subtilisin. HIV-1 GP120 (anti-gp120), Gag p55 (anti-Gag-CAp24), and endogenous CCDC8 (anti-CCDC8) were monitored by Western blot. (**c**) Schematic diagram of wild type Gag (WT) and mutant Δ8-126 (upper panel) and Western blot analysis of wild type VLPs and mutant Δ8–126 (anti-Gag-CAp24) and endogenous CCDC8 (anti-CCDC8) (lower panel). (**d**) Western blot analysis of S1, S100, and P100 co-transfected with HIV-1 BH10 and EGFP or CCDC8 in HEK293T cells. (**e**) Samples of P100 from panel (**d**) were subjected to membrane flotation assay and analyzed by Western blot with anti-Gag (anti-Gag-CAp24), anti-Caveolin-1, anti-TFR and anti-His.

Next, we examined the ability of endogenous CCDC8 to be packaged into HIV-1 viral particles. Endogenous CCDC8 is incorporated into both Gag VLPs and protease-negative BH10 P- (Fig. 2a). In protease-positive BH10 virus, CCDC8 is processed, probably by viral protease (Fig. 2a). We also asked whether CCDC8 is on the virion surface or within the virion particles. Isolated BH10 P- virions were exposed to subtilisin protease before lysis (Fig. 2b). Subtilisin does not penetrate the viral membrane, but processes gp120 and any contaminant proteins present on the external side of the viral membrane. These results confirm the presence of CCDC8 within the virions (Fig. 2b). To further map the region of interaction between Gag and CCDC8, we constructed a Gag mutant lacking all but the first 8 amino acids in matrix (Fig. 2c). This mutant keeps the myristoylation signal at the N terminus of Gag and can still bind to membrane and produce extracellular Gag VLPs. In Fig. 2c, Western blots of viral lysates probed with either anti-Gag or anti-CCDC8 show that endogenous CCDC8 is no longer found in VLPs containing the matrix deletion in Gag, suggesting that CCDC8 may interact with the matrix region in Gag.

### CCDC8 is acting on the post-translational step of HIV-1 replication

CCDC8 causes a strong reduction in virus production (Fig. 1a) that is associated with a much weaker or no reduction in the production of cellular Gag (Fig. 1a–d). This suggests that a major cause of reduced virus production is posttranslational, between Gag synthesis and virion release.

We next examined whether CCDC8 could prevent Gag binding to the membrane for assembly. Cell lysates expressing HIV-1 with CCDC8 or with the control plasmid EGFP were subjected to Dounce homogenization and centrifuged at low speed to remove nuclei and unbroken cells, and the subsequent supernatant (S1) was further ultra-centrifuged, resulting in supernatant (S100) and pellet (P100). Membrane-associated Gag is enriched in P100 (Fig. 2d). Despite CCDC8 overexpression, the Gag in P100 does not significantly change compared to the control (compare the middle lanes in Fig. 2d). We further separated the P100 through membrane flotation sucrose gradient. The pattern of p55 Gag in each section of sucrose gradient from top to bottom has no apparent alteration either, despite the overexpression of CCDC8 or not (Fig. 2e). The membrane-associated Gag mainly exists in the section 2 and 3, as indicated by the membrane markers Caveolin-1 and transferrin receptor (TFR) (Fig. 2e). This suggests that overexpression of CCDC8 does not prevent Gag binding to the membranes, which include internal membrane and plasma membrane. Interestingly, CCDC8 also co-exists with membrane marker Caveolin-1 and TFR in the section 2 and 3 in the sucrose gradient (Fig. 2e).

**Figure 3:**
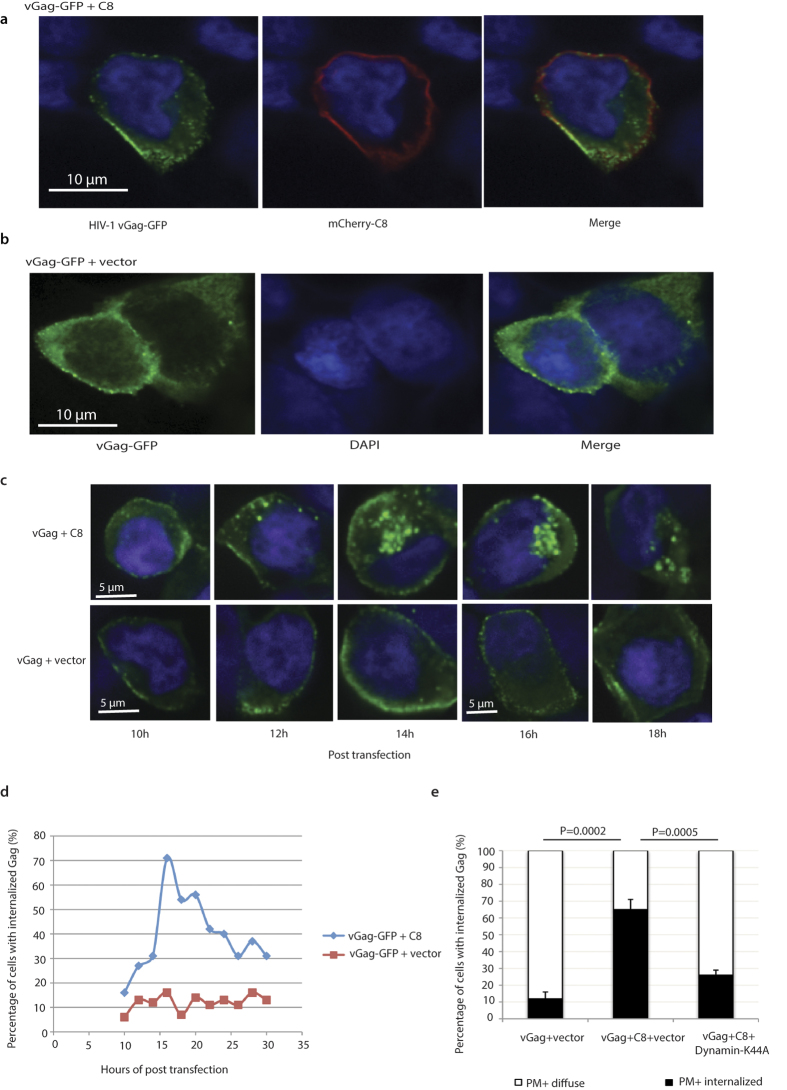
CCDC8 causes internalization of HIV-1 Gag. (**a**) Localization of CCDC8 on the plasma membrane. vGag-GFP (green) and m-Cherry tagged CCDC8 (red) were co-expressed in HEK293T cells and stained by DAPI and observed under confocal microscope. The scale bar represents 10 μm. (**b**) Same as panel (**a**) except vGag-GFP (green) was co-expressed with the empty vector. (**c**) Representative immunofluorescence images of HEK293T cells, which were co-transfected by vGag-GFP and His-tagged pTT5-SH5-CCDC8 or empty vector and then fixed, stained with DAPI, and observed under confocal microscope at the indicating times. (**d**) Quantitative imaging analysis. Ten random fields of HEK293T cells were counted, and the percentage of cells with internalized Gag was calculated and plotted at the indicating time. (**e**) Quantitative analysis of the percentage of cells with internalized Gag at 16 hours post transfection with vGag-GFP, pTT5-SH5-CCDC8, and empty vector or dominant negative dynamin K44A. White bar represents the percentage of cells in which vGag-GFP was localized on the plasma membrane (PM) or diffused in cytoplasm (diffuse). Black bar stands for the percentage of cells with internalized Gag or on the plasma membrane (PM). Error bar represents values ranges from the three independent experiments. Statistical analysis of two-tailed Student T test was performed, P value is indicated.

To further confirm the localization of CCDC8, we used confocal microscopy. Figure 3a clearly shows that mCherry-tagged CCDC8 (red) localizes on the plasma membrane, where Gag assembles (green) (vGag-GFP, NIH AIDS Research Reagent Program, Catalog number 11468, from Dr. Andrew Mouland). With or without HIV-1 Gag, CCDC8 is mainly present on the membrane, and only a small part is present in the cytoplasm (Fig. 3a and unshown data).

### CCDC8 causes an internal accumulation of Gag particles in the cytoplasm

Comparing the appearance of vGag-GFP co-expressed with CCDC8 or with empty vector (Fig. 3a,b), we found that for cells expressing vGag-GFP and empty vector, vGag-GFP is present homogeneously in the cytoplasm, representing Gag monomer or low-order Gag multimers, and on the plasma membrane as bright vGag-GFP puncta, representing high-order Gag multimers or assembly particles (Fig. 3b)^12^. When Gag and CCDC8 are co-expressed, there is an accumulation of bright vGag-GFP puncta (see the cytoplasm in the cell in Fig. 3a left panel) at 24 hours post-transfection, indicating that CCDC8 probably changes Gag trafficking. There are two possible explanations for the data; one is that Gag is not transported to the plasma membrane, but directly accumulates in the intracellular sites; the other is that Gag is first transported to the membrane but is re-internalized, or redirecting to the intracellular sites. The membrane flotation sucrose gradient data, which show that CCDC8 does not prevent Gag binding to the membrane, support the latter possibility.

### Internalization of Gag from the plasma membrane results from endocytosis

To examine the kinetics of Gag accumulation, we traced the vGag-GFP at various time points after transfection. The transfected HEK293T cells were fixed and observed under confocal microscopy. At 10 hours after co-transfection of CCDC8 and vGag-GFP, around 84% of the cells containing vGag-GFP showed exclusively Gag localization at the plasma membrane. Only ~16% of the cells displayed intracellular accumulation of vGag-GFP puncta (Fig. 3c,d). As time passing, more cells presented intracellular vGag-GFP puncta accumulation. At 16 h post transfection of CCDC8 and vGag-GFP, the cells with intracellular vGag-GFP puncta accumulation peaked at ~71%, and then declined slowly (Fig. 3c,d). Notably, the visible accumulation of vGag-GFP puncta at the plasma membrane significantly preceded its accumulation in intracellular sites (Fig. 3c,d), suggesting that Gag is first transported to the plasma membrane and subsequently re-internalized. In contrast, upon co-expression of vGag-GFP and empty vector, the proportion of cells with intracellular vGag-GFP puncta accumulation remains constant throughout time at roughly 10% (Fig. 3c,d).

Because previous data showed that Gag assembly occurs on the plasma membrane^12^, these data suggest that CCDC8 changes Gag trafficking. Namely, after Gag meets CCDC8 on the plasma membrane, Gag is internalized instead of budding out. This is in accordance with the membrane flotation data that Gag still binds to the membrane upon overexpression of CCDC8 (Fig. 2e). It is possible that Gag localizes to the membrane because of a low level of CCDC8 at the beginning of transfection. To test this, we performed CCDC8 transfection 10 hours earlier than Gag transfection to allow for a CCDC8 build-up (Supplementary Figure 1a). The scenario appeared again that visible accumulation of vGag-GFP puncta at the plasma membrane significantly preceded its accumulation in intracellular sites (Supplementary Figure 1a). These data support the notion that Gag internalization is facilitated by its interaction with membrane-associated CCDC8. To further confirm CCDC8-mediated Gag internalization, we co-expressed CCDC8 and vGag-GFP with dominant negative Dynamin-K44A, an endocytosis inhibitor. Dynamin-K44A significantly reduced the CCDC8-mediated Gag internalization (P = 0.0005, Fig. 3e, Supplementary Figure 1b), suggesting that CCDC8-mediated Gag internalization results from endocytosis. It appears that the meeting of Gag with CCDC8 on the plasma membrane is a prerequisite for Gag endocytosis.

Virion endocytosis usually goes into endolysosome pathway and virions always accumulate in endolysosomes. Thus, we determined whether CCDC8 induced this phenotype. vGag-GFP was co-expressed with His-tagged CCDC8, and cells were fixed and stained with antibody against CD63, an endolysosome marker. Supplementary Figure 2a shows that the CCDC8-mediated internalized vGag-GFP does not colocalize with CD63 (3D structure, Pearson’s colocalization coefficient ~0.1). This data suggest that CCDC8-mediated endocytosis of Gag does not go into endolysosome pathway.

### Gag undergoes CCDC8-dependent polyubiquitination and degradation

**Figure 4:**
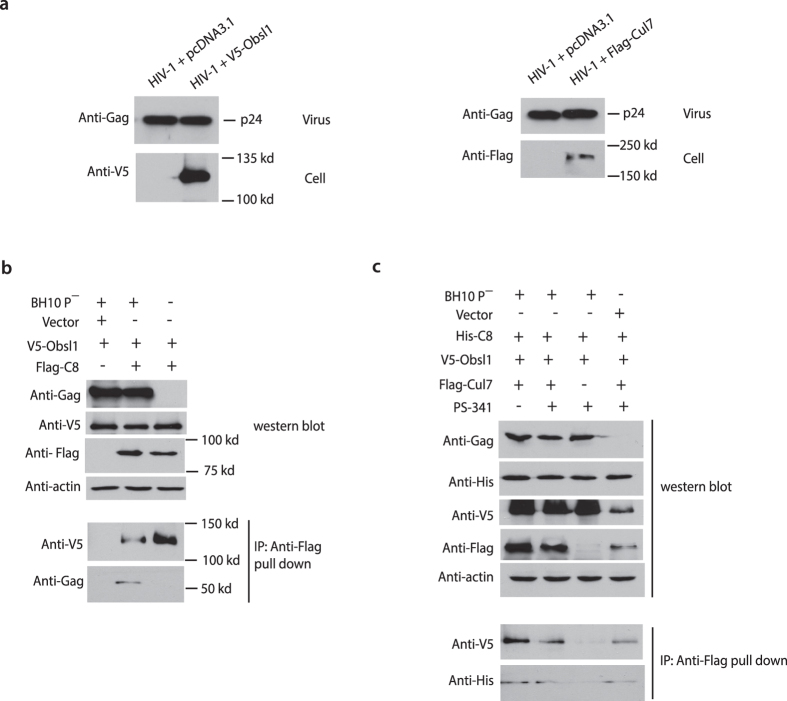
Interaction between Gag, CCDC8, Obsl1, and Cul7. (**a**) Western blot analysis of the effect of Obsl1 and Cul7 on the production of HIV-1. Obsl1 is tagged V5 and Cul7 is tagged Flag. (**b**) Western blot analysis of the interaction between Gag, CCDC8, and Obsl1 by co-immunoprecipitation. (**c**) Western blot analysis of the interaction between Gag, CCDC8, Obsl1, and Cul7 from co-immunoprecipitation assay. Proteasome inhibitor PS-341 was added.

Since the reported role of CCDC8 mutations in 3-M syndrome links it to Obsl1 and E3 ligase Cul7, we tested whether Obsl1 or Cul7 could also inhibit HIV-1 production. Unlike CCDC8, co-expression of HIV-1 with Obsl1 or Cul7 does not inhibit the production of Gag CAp24 (Fig. 4a).

Next, we investigated possible interactions between Gag, CCDC8, Obsl1, and Cul7. Co-immunoprecipitation of exogenously expressed Gag, V5-tagged Obsl1, and Flag-tagged CCDC8 showed interaction between Gag and CCDC8, and interaction between CCDC8 and Obsl1, but not in the negative control (Fig. 4b). This is further evidence that Gag can interact with CCDC8, aside from the incorporation of CCDC8 into the HIV-1 virions and localization on the plasma membrane. Co-immunoprecipitation also demonstrated the interaction between Cul7, Obsl1, and CCDC8 (Fig. 4c).

**Figure 5:**
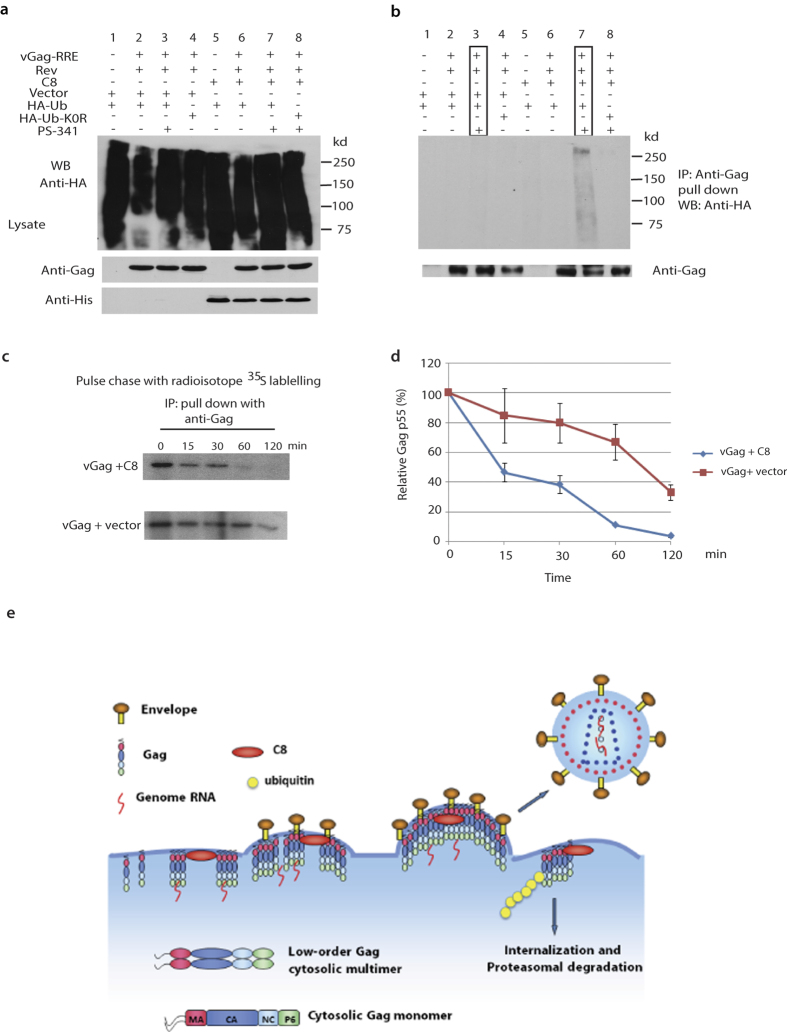
CCDC8-mediated Gag polyubiquitination and degradation. Western blot analysis of cell lysates (left panel) (**a**) and co-immunoprecipitation of cell lysate (right panel) (**b**) with anti-Gag, anti-His and anti-HA. (**c**) Pulse chase experiment with radioisotope ^35^S labelled methionine and cysteine. (**d**) The data in panel (**c**) were plotted. Error bar represents value ranges from three independent experiments. (**e**) Model of HIV-1 inhibition by CCDC8.

Since we observed the interaction between Gag and CCDC8, and between CCDC8 and Obsl1 and E3 ligase Cul7, we examined the state of Gag ubiquitination upon exposure to CCDC8 (Fig. 5a,b). Viral Gag-RRE, His-CCDC8, and a plasmid coding for haemagglutinin- tagged ubiquitin (HA-ubiquitin) were transiently co-transfected in HEK293T cells. In some transfections, cells were also transfected with HA-Ub-K0R, a mutant of ubiquitin that contains substitutions for K48R and K63R, two important lysine amino acids mutated to arginine^13^. 24 hours post-transfection, some cells were also exposed to the proteasome inhibitor, PS-341, for an additional 12 hours, followed by cell lysis, co-immunoprecipitation and Western blot analysis. The results show that ubiquitination of cell lysate proteins occurs under all the used conditions, including the mutant HA-Ub-K0R (Fig. 5a). On the other hand, after treatment of cells with proteasome inhibitor PS341 in co-immunoprecipitation, a strong polyubiquitination signal of immunoprecipitated Gag is detected upon overexpression of CCDC8 (Fig. 5b lane 7), contrary to control cells expressing the empty vector (Fig. 5b lane 3). Previous work has shown the mono- and di-ubiquitinated Gag, associated with viral budding, migrates approximately 55–72 kd in SDS-PAGE gel^14^, which was successfully repeated by us (unshown data). Compared to the polyubiquitination, mono- or di-ubiquitination of Gag is too weak to be seen (Fig. 5b). Gag polyubiquitination does not occur when Gag does not present (lanes 1, 5), nor the overexpression of CCDC8 (lanes 2–4). Polyubiquitination also does not occur when HA-Ub-K0R replaces HA-Ub, suggesting a requirement for K48 or K63-dependent polyubiquitination (lane 8). Lane 7 in Fig. 4b indicates that the proteasome inhibitor PS-341 is required to be able to detect polyubiquitinated Gag (compare lane 6 and 7).

Next, we checked the metabolic half-life of Gag upon overexpression of CCDC8 using a pulse-chase experiment. The culture medium was complemented with the same amount of radioisotope ^35^S-labeled methionine and cysteine, and Gag in cell lysate was chased using immunoprecipitation at the indicated times (Fig. 5c,d). Normally, HIV-1 Gag will bud out and ^35^S-labled Gag will decrease over time. CCDC8 apparently reduced the half-life of Gag protein (Fig. 5c,d). The half-life of Gag upon overexpression of CCDC8 is around ~20 minutes, compared to ~80 minutes in the counterpart. This indicates that CCDC8 accelerates Gag degradation.

## Discussion

This study identifies a new virion-incorporated protein, CCDC8, and its associated pathway that inhibits HIV-1 assembly, and triggers HIV-1 Gag endocytosis, polyubiquitination, and degradation. CCDC8 is a membrane- associated protein, and exogenous overexpression of CCDC8 results in a strong reduction in HIV-1 virion release.

Different techniques were used in this study, including Western blot, imaging test, immunoprecipitation and pulse-chase test. All these data were in consistence and suggested the conclusion of this study. In immunofluorescence test, Gag was tagged GFP and CCDC8 was tagged mCherry. The bright Gag-GFP puncta represent high-order Gag multimers or Gag assembly complexes because of the intensity of their fluorescent signal. The loss of extracellular HIV-1 Gag production appears to be due to their internalization by endocytosis, as demonstrated in Fig. 3e using the endocytic inhibitor, Dynamin-K44A. The loss may be due to CCDC8’s interaction with the matrix region in Gag, which is responsible for the incorporation of CCDC8 into the virions. Moreover, inhibition of Gag assembly by CCDC8 is dependent on its elevated expression in a dose-dependent manner, since millions of Gag proteins bind to the plasma membrane for assembly. Gag proteolytic processing to release CAp24 occurs after the Gag arrival to the plasma membrane for assembly^1^,^15^. This explains an observed rapid decrease in CAp24, and a slow decrease in p55 Gag in the cell lysate when CCDC8 was co-expressed with HIV-1 (Fig. 1a,b). The apparent inhibition of CAp24 production in the cell lysate by CCDC8 is more likely a second effect of assembly inhibition.

Endocytosis refers to a process of invagination of the cargo protein and cell membrane to form a pocket, internalization and membrane scission^16^,^17^. Clathrin-dependent endocytosis is typical. However, ubiquitin-dependent endocytosis has also been described; for example, ubiquitin-mediated endocytosis has been reported for the membrane-bound epidermal growth factor (EGF) receptor (EGFR). When stimulated with low-doses of EGF, EGFR is internalized almost exclusively through the clathrin-dependent pathway, while at higher concentrations of EGF, endocytosis of EGFR is through a clathrin-independent, ubiquitin-dependent pathway^18^,^19^. This also implies the possibility that CCDC8-mediated Gag polyubiquitination could be the triggering signal for Gag endocytosis.

In this study, we report that Gag is polyubiquitinated, even though mono- and di-ubiquitination of Gag p6 for promoting virus budding have been reported^14^,^20^. Dr. Ulrich Schubert group reported Gag polyubiquitination in Gag p6 mutants S40F^21^ and deltaPTAP^14^, but those were not wild-type Gag. When this manuscript was in preparation and submission, Nityanandam reported that BCA2/Rabring7 targets Gag and induces Gag polyubiquitination^22^. In this study, Gag polyubiquitination is CCDC8-dependent (Fig. 5), and associated with the CCDC8 pathway proteins^10^,^11^. Due to the association of CCDC8 with the plasma membrane (Figs 2 and 3), we theorize the following scenario: multimer Gag assembly units interact with CCDC8, Obsl1, and Cul7 at the plasma membrane, causing Gag polyubiquitination. The polyubiquitination signal might trigger Gag endocytosis, and then Gag complex enters into 26S proteasome, resulting in an eventual degradation of the complex by the ubiquitin-proteasome system (Fig. 5e model). However, we still cannot exclude another possibility that polyubiquitination of Gag occurs in the cytoplasm, since Cul7 localizes in the cytoplasm too. The relationship between CCDC8-mediated Gag polyubiquitination and endocytosis is still elusive.

It is unknown what mechanism underlies the growth-related disorder 3-M syndrome. The serum growth hormone (GH) level of 3-M patients is usually normal, and insulin-like growth factor 1(IGF-1) is normal or low^10^. An intriguing recent finding is that Cul7-Fbw8 can degrade the insulin receptor substrate 1 (IRS-1) through Skp1-Fbw8-ROC1 (SCF complex), suggesting the mechanism of 3-M syndrome is related to IRS-1^23^. As well, transcription microarray data showed that the insulin-like growth factor 2 (IGF-2) is down-regulated in 3-M patients^24^. Therefore, we speculate that 3-M syndrome is related to a disorder of insulin-like growth factor or receptor signaling. Interestingly, another 3-M related protein, insulin-like growth factor II mRNA binding protein 1 (IMP1, or IGF2BP1), was reported to inhibit HIV-1 assembly^25^,^26^. It may be informative to investigate more generally the potential of 3-M syndrome pathway proteins in the inhibition of HIV-1 assembly.

Endogenous expression of CCDC8 is similarly low in many human tissues. In some cell lines, such as Jurkat T lymphocyte and HEK293 cells, the endogenous expression is a little higher (GeneCard, http://www.genecards.org/cgi-bin/carddisp.pl?gene=CCDC8). Apparently, no human cells or cell lines endogenously express sufficiently high levels of CCDC8 to accomplish HIV-1 inhibition. Using siRNA technique, we down-regulated the expression of CCDC8, and found that HIV-1 Gag production was not significantly or modestly altered in HEK293T cells. Therefore, this study is not able to address the role of endogenously expressed CCDC8.

However, it is well accepted that proteasomal degradation of pathogen-derived proteins plays a key role in antigen presentation by MHC class I molecules^14^. Intriguingly, CCDC8 inhibits the viral Rev-RRE source native-codon Gag assembly, but not humanized or synthesized-codon Gag. Namely, CCDC8 can distinguish between “self” and “non self” HIV-1 Gag (unshown data). This mechanism is central to immune system recognition of pathogens. It is possible that the human immune system can “sense” HIV-1 attack by CCDC8-medicated Gag proteasomal degradation to present antigens to the immune system.

The CCDC8 gene is highly conserved among mammalian species, including human, chimpanzee, rhesus monkey, dog, cow, mouse, and rat (http://www.ncbi.nlm.nih.gov/gene/83987). CCDC8 was also reported to be a cofactor required for p53-mediated apoptosis following DNA damage in humans^27^. Therefore, CCDC8 is a very important factor relevant to cell growth, apoptosis, and probable anti-viral infections, although its exact functions are unknown. Further work is needed for clarifying its roles in all of these cellular processes.

In conclusion, this finding sheds light on a new pathway for inhibiting HIV-1 assembly. It presents a potential target for pharmaceutical drug development against HIV-1.

## Methods

All experiments were carried out in accordance with protocols approved by Ethics Committee in Lady Davis Institute, McGill University and China Center for Disease Control and Prevention.

### Cells and plasmids

HEK293T (CRL-11268, ATCC), Hela-TZM-bl (NIH AIDS Research Center, catalog no. 8129) cells were cultured in Dulbecco’s Modified Eagle Medium supplemented with 10% fetal bovine serum.

Plasmid of vGag-RRE was constructed by PCR. HIV-1 BH10 RRE was amplified using primers (5′ GCGCCTCTAGATAAATTGAACCATTAGGAGTAGC 3′, HXB2 7680–7714, 5′ GAGTGGGCCCATTACTCCAACTAGCATTCCAAGG 3′, HXB2 8064–8049) and cloned into pcDNA3.1 (Invitrogen, USA) XbaI and ApaI sites. Cleavage sites of restriction endonuclease are underlined. HIV-1 BH10 Gag with 5′ LTR was amplified by (5′ GATCGAATTCGGTCTCTCTGGTTAGACCAGATCT 3′, HXB2 445–478, 5′ GACTCTCGAGTTACGTTATTGTGACGAGGGGTCG 3′, HXB2 2307–2274) and cloned into EcoRI and XhoI sites of above vector. vGag/GagPol-RRE was a gift from Dr. Chen Liang (McGill University, Canada). vGag/GagPol-RRE P- (D25G) was introduced by PCR (5′ GGAAGCTCTATTAGGTACAGGAGCAGATGATAC AG 3′, 5′ CTGTATCATCTGCTCCTGTA CCTAATAGAGCTTCC 3′) (QuikChange Site-Directed Mutagenesis Kits, Stratagene, USA) and confirmed by sequencing. CCDC8 was amplified and cloned into pTT5-SH5 vector BamHI and EcoRI sites (5′ GATACTGGATCCCTGCAGATCGGGGAGGACGTC 3′, 5′ GATCAT GAATTCTCACAGCTGGTCTTCTTGC TCTCC 3′). V5-Obsl1 is a kindly gift from Dr. Graeme Black (University of Manchester, Manchester, UK). Flag-Cul7 is also a gift from Dr. Zhen-Qiang Pan (The Mount Sinai School of Medicine, New York, USA).

### Mass Spectrometry analysis

The viral lysates were resolved by SDS-PAGE gel and stained by Coomassie-blue. Samples were cut into small pieces and be analyzed by nanoliquid chromatography/tandem mass spectrometry (nano-LC-MS/MS) in Institute For Research in Immunology and Cancer (IRIC), University of Montreal, Montreal, Canada.

### Membrane flotation analysis

Membrane flotation analysis is performed as previously described^28^. Briefly, forty-eight hours posttransfection, the cells were treated with hypotonic Tris-EDTA (TE) buffer (20mM Tris-HCl ph7.4, 1 mM EDTA, 0.01% β-mercaptoethanol) supplemented with a protease inhibitor cocktail (Roche). After Dounce homogenization, the cell homogenate was then centrifuged at 1500 g for 30 minutes to remove the nuclei and unbroken cells. The supernatant (S1) was then centrifuged for 1 h at 100,000 g in SW55Ti rotor (Beckman) at 4 °C, resulting in the supernatant (S100) and the pellet (P100). The P100 was resuspended in TNE buffer (20 mM Tris-HCl ph 7.5, 100 mM NaCl, 1 mM EDTA). A total of 200 μl of each lysate was mixed with 1ml of 85.5% sucrose (w/v) in TNE buffer, placed at the bottom of ultracentrifuge tubes, and overlaid with 2.5 ml of 65% sucrose and 1.5 ml of 10% sucrose. The samples were centrifuged at 4 °C in a SW55Ti rotor for 16 hours at 35,000 rpm. Nine equal fractions were collected from the top to the bottom.

### Antibodies

We listed all antibodies used in this study, anti-Gag (made by ourselves); anti HIV-1 Env gp120 (Abcam); anti-CCDC8 (Cat#: H00083987-B01P, Abnova); anti-Caveolin-1 (Santa Cruz Biotechnology); anti-TFR (Invitrogen); anti-His (Invitrogen), anti-Flag (Invitrogen), anti-HA (Invitrogen), anti-V5 (Invitrogen), anti-CD63 (Abcam), anti-26S proteasome marker PSMD8 (Cat #: H00005714-B01, Abnova) and secondary antibody anti-mouse (Invitrogen), anti-rabbit (Invitrogen).

### Microscopy

HEK293T cells were cultured on glass coverslips and transfected with plasmids as shown in the figures. After the indicated time posttransfection, the cells were fixed in 4% paraformaldehyde for 5 min and followed by cold methanol at −20 °C for 15 min. The cells stained with or without antibody, and DAPI, and examined using Zeiss confocal microscope.

### Immunoprecipitation

After 48 hours posttransfection, the cells were lysed on the ice with BC100 buffer (20 mM Tris-HCl, pH7.5, 100 mM NaCl, 10% glycerol, 0.2 mM EDTA, 1% Triton X-100 and 1% CHAPS supplemented with protease inhibitor tablets), as described previously with a little modification^27^. The lysates were incubated with monoclonal Anti-Flag M2-agorose beads (Catalog A2220, Sigma-Aldrich) at 4 °C overnight. After five time washes with Flag buffer (50 mM Tris-HCl, pH 7.5, 150 mM NaCl, 10 mM NaF, 1 mM EDTA, 1% Triton X-100, 1% CHAPS, 0.2% sarkosyl, 10% glycerol), the beads were eluted with 3×Flag peptide (Catalog F4799, Sigma-aldrich), and then resolved by SDS-PAGE and analyzed by Western blot.

### *In vivo* ubiquitination assay

HEK293T cells were transfected with vGag-RRE, Rev, HA-Ubiquitin or HA-Ubiquitin-K0R (Ubiquitin mutant containing K48R and K63R), vector or CCDC8. At 24 h posttransfection, 50 nM PS-341 (Proteasome Inhibitor, Catalog 1846, BioVision), was added to inhibit the degradation of Gag. After another 12 hour, the cells were lysed at room temperature with detergent-rich RIPA buffer (50 mM Tris-HCl pH 7.4, 150 mM NaCl, 1 mM EDTA, 1.0% glycerol, 0.5% SDS, supplemented with protease inhibitor tablets (Roche) and 5mM N-ethylmaleimide (ETM222.1, Bioshop Canada) to inhibit deubiquitination as described previously^29^,^30^, and cleared by centrifugation at 10,000 g for 30 min at 4 °C. The supernatants were then diluted 5-fold in the same buffer containing NP-40 rather than SDS, to adjust the concentration of SDS to 0.1% and NP-40 to 1.0%. Immunoprecipitation was performed with anti-Gag antibody and Protein A Sepharose beads (CL-4B, Catalog 17-0780-01, GE healthcare). The beads were washed twice with above buffer containing 1.0% NP-40 and eluted with elution buffer (0.1 M glycine, pH 2.0), followed by SDS-PAGE separation and Western blot analysis^29^.

### Metabolic labelling and pulse chase experiment

HEK293T cells were transfected with vGag-RRE, Rev, empty vector or CCDC8. Two days post-transfection, the cells were washed with PBS, and starved for 30 minutes in methionine-cysteine free medium supplemented with 2% dialyzed fetal bovine serum (Invitrogen). Then, the cells were labeled for 20 min in medium containing 200 μCi [^35^S] methionine and cysteine (Express [^35^S] Protein Labelling Mix, NEG07200, Perkin Elmer Life Sciences). The pulse was ended by adding prewarmed DMEM supplemented with 10% fetal bovine serum and 5 mM of unlabeled methionine and cysteine. After various chase time periods, the cells were pelleted on ice and washed with ice cold PBS. For immunoprecipitation, the cells were lysed, and Gag was immunoprecipitated with anti-Gag antibody. The proteins were separated by 10% SDS-PAGE gel. The gel was fixed by buffer containing 40% ethanol, 10% acetic acid, 10% glycine at room temperature for 2 hours and amplified (NAMP 100, GE life Science) for 15 minutes, dried the gel and put the film on −80 °C freezer, then quantified by ImageJ.

## Additional Information

**How to cite this article**: Wei, M. *et al.* Inhibition of HIV-1 assembly by coiled-coil domain containing protein 8 in human cells. *Sci. Rep.* **5**, 14724; doi: 10.1038/srep14724 (2015).

## Supplementary Material

Supplementary Information
