# Supplementary material for: Inhibition of HIV-1 assembly by coiled-coil domain containing protein 8 in human cells
Source: Sci Rep. 2015 Oct 1;5:14724. doi: 10.1038/srep14724 (PMC4589731; doi:10.1038/srep14724)
Supplement: Supplementary Information [file srep14724-s1.pdf]

Inhibition of HIV-1 assembly by coiled-coil domain containing protein 8 in human cells

Min Wei, Xia Zhao, Mi Liu, Zhi Huang, Yong Xiao, Meijuan Niu, Yiming Shao, Lawrence Kleiman

Supplementary figures

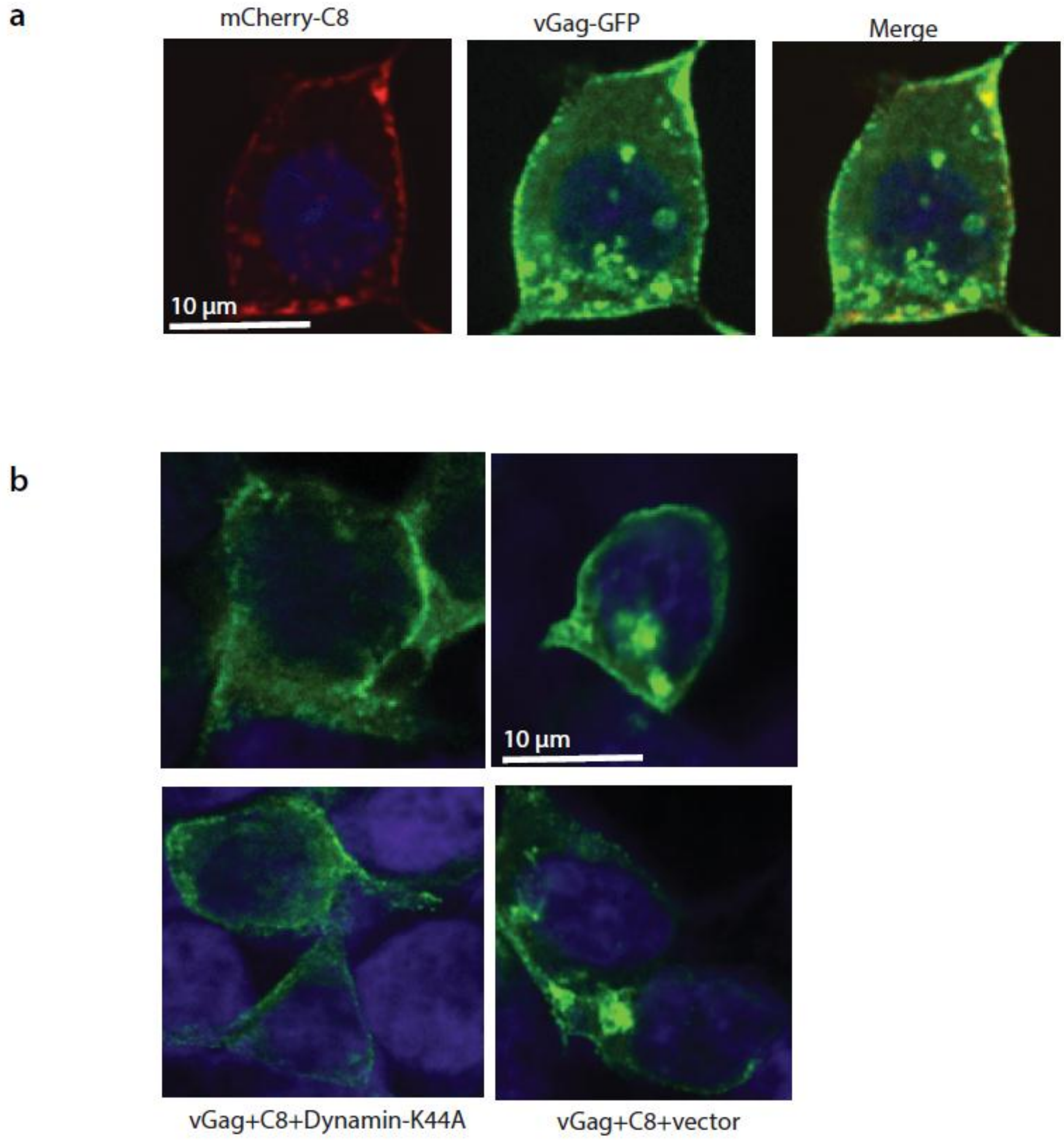

Supplementary Figure 1. CCDC8 causes internalization of HIV-1 Gag.
(**a**) Representative immunofluorescence images. mCherry tagged CCDC8 (mCherry-C8) (red) was transfected 10 hours earlier than vGag-GFP in HEK293T cells. 24 hours after the vGag-GFP transfection, the cells were fixed and observed. (**b**) vGag-GFP, pTT5-SH5-CCDC8, and empty vector or dominant negative dynamin K44A were co-expressed in HEK293T cells, and then the cells were fixed at 24 hours and observed. Two pictures are shown for each experiment.

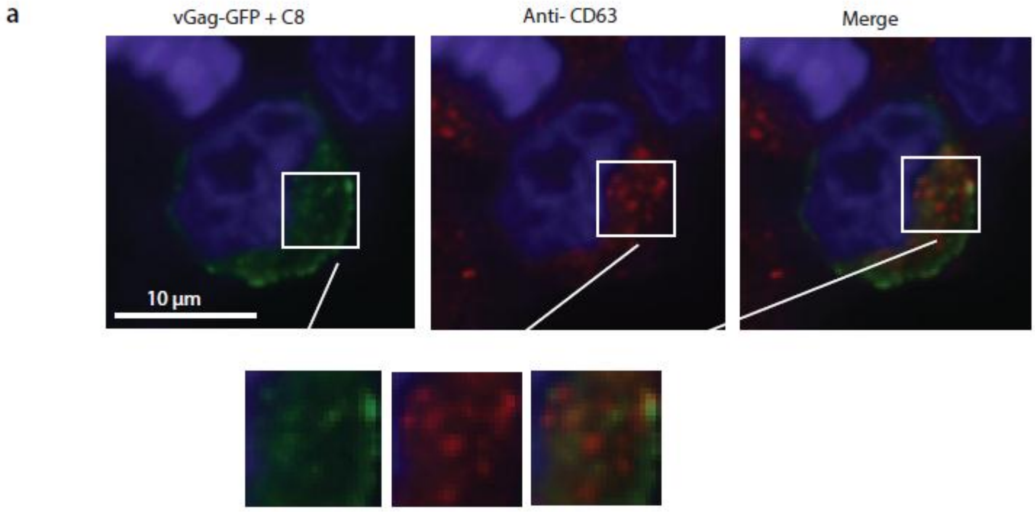

Supplementary Figure 2. CCDC8-mediated endocytosis of Gag does not go into endolysosome pathway. (**a**) Representative immunofluorescence images of co-expressed vGag-GFP (green) and pTT5-SH5-CCDC8 in HEK293T cells. At 24 hours post transfection, the cells were fixed and stained with DAPI (blue) and anti-CD63 (red), an endolysosome marker, and followed by the secondary Alexa fluor 594-conjugated antibody (Invitrogen).
